# Cases of Strangulated Hernia

**Published:** 1827-07

**Authors:** H. Earle

**Affiliations:** St. Bartholomew's Hospital.


### STRANGULATED HERNIA.

*Cases of Strangulated Hernia, operated on by* Mr. H. Earle, *at* St. Bartholomew's Hospital.

The almost endless variety presented by cases of strangulated hernia occurring in different parts of the body, renders a brief record of almost every instance operated on of importance to the surgeon; more especially when such varieties involve any practical question of moment. Two cases of this description occurred lately at St. Bartholomew's, some particulars of which may deserve to stand upon record. They were both cases of what is usually termed congenital hernia: that is to say, the tunica vaginalis of the testis formed the hernial sac. The term "congenital" was however very inapplicable to these, as indeed it is to most of such cases: in one the hernia did not descend until after the age of twelve, and in the other not till between seven and eight. With a view to obtaining a more correct nomenclature, I would venture to suggest the propriety of calling such herniæ "Scroto-vaginal."

Case I.—James Ellis, a powerful, healthy man, aged thirty, was admitted, April 27th, at twelve o'clock, with a large scrotal hernia, of which he gave the following history:—The hernia first occurred at the age of twelve, while serving on board a ship. It was produced by a fall, and violent blow on the abdomen. The testicle had been down from his earliest childhood. The hernia was reduced, and he procured a truss, which he used until it was worn out. He omitted to wear it for ten years, during which time the hernia only came down occasionally, though he was actively employed, and often exerted himself, as a brewer's servant, to his utmost. During the last four years, the hernia had become more troublesome, and obliged him to wear a truss constantly. At about half-past nine of the morning of his admission, the rupture protruded behind the truss, without any unusual exertion having been made. He was not able to return it, and it rapidly increased to a large size, and became very tense and painful to the touch.

I saw the patient about half past twelve, and found that some attempts had been made to reduce it before and after his admission. He was placed in a hot bath, and bled to the extent of twenty-eight ounces, when he became rather faint and disposed to be sick. The taxis was resorted to whilst in the bath, and immediately on his removal; but no effect could be produced on the bulk of the tumor, which was too tender to admit of being much handled. The tobacco enema was next directed: two half-drachms were thrown up after a short interval, which produced considerable depression of the pulse and sickness; under which circumstances the taxis was again employed for a few minutes, when the increasing tenderness of the tumor and abdomen called for an immediate operation, which was performed soon after two o'clock.

The tumor was very large, and distended the scrotum; the testicle could be felt at the back of the tumor, but could not be separated from it, which led me to consider the case as scroto-vaginal. The sac contained much fluid, and a large fold of small intestine, about seven inches in length, much discoloured, and very closely girt at the external ring. Between the two extremities of the intestine, a portion of thickened omentum, with its vessels gorged with blood, was protruded, and greatly increased the stricture on the gut. When the sac was fairly laid open, and the edge of the external ring divided with the hernial knife, not the slightest impression could be made on the protruded gut and omentum, nor could any fresh portion be drawn down.

The abdominal canal was next laid open, the sac closely investing the protruded part throughout its extent. An additional portion of healthy intestine could now be brought down; but the stricture at the inner ring was so narrow, that a *third* application of the hernial knife was rendered necessary.* The omentum was now returned, and a considerable portion of healthy gut drawn down, in the hopes of diminishing the volume of the protruded part, which was greatly distended. The reduction of the whole was, on this account, rather tedious. The testis was seen at the bottom of the sac, enveloped with its tunica albuginea. The vaginal coat was not divided to its lower extremity, so that the testis retained its proper covering, and no hernia humoralis ensued.

The edges of the wound were brought together with three sutures; a compress of wet lint and a bandage completed the dressings.

After the operation, a purgative enema was thrown up to clear the large intestines; and Magnesiæ Sulphatis ʒss. was ordered to be taken every three hours.

* I mention this circumstance, the rather as in a publication, which professes to give correct Hospital Reports, it is stated that the hernial knife was not used at all!! Lancet ?

At five P.M. he complained of slight pain over the abdomen, and his pulse was full and hard. He was bled to the extent of twenty-four ounces, which much relieved him; and he passed a feculent stool. At eight P.M. his pulse was soft and skin moist, and he expressed himself as quite comfortable, and anxious to sleep.

The following day he took some castor-oil, to which he had been accustomed to resort when constipated. This procured copious evacuations, and from that time he never had a bad symptom. The wound healed by the first intention, except at one small spot.

This case is powerfully illustrative of the importance of operating early. Not six hours had elapsed from the time of the descent, yet so rapidly had strangulation taken place that it was not possible to reduce the hernia, even when the sac was opened, without freely dividing the canal and internal ring, or rather the neck of the sac. How perfectly unavailing the taxis must be in such a case, is too obvious to need a comment. A very few hours' delay in trying other remedies would probably have proved fatal, as the gut was gorged with blood, and greatly distended. Its peritonial surface was, however, smooth and healthy. In this case the tunica vaginalis was much dilated in its whole extent from the external ring, and the testis occupied its natural situation in the scrotum. It affords an illustration of the late period at which this description of hernia may take place, when the communication remains open after the descent of the testicle.

Case II.—Robert Matthews, aged twenty-nine, a strong, healthy man, was admitted into the hospital, soon after midnight, on the 1st of May, with strangulated hernia, of which he gave the following account:—When seven or eight years old, the testicle on the right side passed through the external ring, but never descended so low as the other testicle. Soon after this time it was discovered that he had a rupture on that side, for which a truss was applied; but, in consequence of its pressing on the testicle, he was unable to wear it. The rupture had remained nearly stationary from that time, and he was always able to reduce it until the preceding night. After having been at the theatre, on his return home he felt pain in the tumor, which was more tense than usual. This continued through the night, and by the morning the size of the swelling was much increased, and he brought every thing off his stomach. He called in a medical gentleman, who gave him several doses of purgative medicine, (Calomel and Jalap, and Infus. Sennæ comp.) He continued to get worse through the day, and at night it was ascertained that his symptoms were connected with the rupture, and he was brought into the hospital.

I saw him about two A.M. and found him suffering severe pain in the tumor, which was very tense, and he complained of much tenderness in the lower part of the abdomen. His pulse was rather feeble and oppressed, his countenance anxious, and his skin clammy. Under these circumstances, I determined on operating immediately. On dividing through the integuments, the sac presented an unusual appearance: it was narrow for about an inch and a half from the ring, and then swelled out into a large globular tumor. On puncturing this, some turbid fluid escaped, which had a fetid smell. This was more sensible after the whole sac was opened, when a large fold of very dark diseased small intestine came into view. The surface of the bowel was rough, and so altered in colour that I entertained serious apprehensions that it had lost its vitality. It was very closely girt by a firm annular contraction at the lower part of the tunica vaginalis of the chord; the sac being formed by that portion which should have enveloped the testicle, and which had assumed the size and shape of a large hydrocele. On slitting up the vaginal canal, which was narrow and much distended, as high as the external ring, I found no impediment at the ring, and ascertained that the opening communicated directly with the abdomen, and not, as in the last case, in an oblique direction. There was, in fact, no distinction between the external and internal rings. The intestine immediately above the stricture, even that portion contained in the vaginal canal, was comparatively quite healthy. Having removed all stricture, a considerable portion of healthy intestine was drawn down; and, by gently compressing the diseased portion, which was very tense, it was nearly emptied into the more healthy bowel. I still hesitated as to the propriety of returning it into the abdomen, as the fetor and colour indicated that sloughing had commenced. After some moments, the bowel began to again distend, and preserved its form, instead of remaining collapsed; and I could now distinctly feel a feeble pulsation in the branches of the mesenteric artery which supplied it. This determined me to give the patient his chance of recovery. I divided the neck of the sac at the ring, to enable me to return the gut with as little force as possible, and slowly and cautiously returned it. The prognosis, under such circumstances, could not be otherwise than most unfavourable. He was directed to have repeated glysters, and, if the pulse rose, to lose blood; but not to take any aperient for some hours, as he had already taken several doses.

At three A.M. his pulse became fuller and stronger, and he had pain in the abdomen. He was bled to the extent of thirty ounces, and his belly was fomented. He had two injections administered, which brought away four stools.

At eight A.M. he complained much of tenderness at the lower part of the abdomen near the hernia, and his pulse was rather hard. He was bled to sixteen ounces, which reduced the pulse, and caused faintness. He took a saline draught, with Magnesiæ Sulph. ℥j. after which he passed four fluid stools. At half-past twelve, his pulse was quick and irritable; tongue and skin moist; and he only complained of pressure in the region immediately above the ring, which was probably occupied by the diseased portion of gut. He could bear pressure on every other part, and expressed himself as feeling very comfortable. He was ordered Cal. gr. iij. Opii gr. j. at night, and a small dose of Castor-oil early in the morning. This procured him a good night, and several decidedly feculent stools in the morning.

From this time his treatment consisted entirely in the repeated application of leeches over the diseased portion of bowel, where there was obviously considerable hardness and pain on pressure, and barley-gruel in very small quantities, to give the stomach and bowels as little duty as possible. Glysters were occasionally thrown up. No aperient medicine was given for many days: and the case went on favourably until the 27th, when he complained of more pain in the seat of the disease, and some tenderness extending over the abdomen; for which he was bled to the extent of sixteen ounces, and twenty leeches applied twice to the abdomen.

On the 29th, I felt an evident fluctuation just above the wound, which had healed, except where a ligature had been applied. On puncturing this with a lancet, about six ounces of good pus came away. The orifice was enlarged, and a bread poultice applied. Matter continued to be discharged for some days; all tenderness in the seat of disease gradually ceased, and the abscess closed finally in about a week. The testicle, which was wasted and flabby, swelled a little after the operation; but this gradually subsided, and it can now scarcely be felt. There is still some induration to be felt just above the incision, occupying nearly the extent and form of the fold of gut. The adhesions which have formed round this will probably render it unnecessary for him to wear a truss; a circumstance of much importance, as, from the situation of the testicle, it would be difficult to construct one which would not distress him. He is still in the hospital, but walking about the ward convalescent.

This case I consider interesting, from the situation of the stricture, which is by no means common. Wrisberg gives a similar case; and in Mr. Lawrence's valuable work on Hernia there is a very analogous case. The termination in this case was far more favourable than could have been expected. In the course of my experience, I never met with an instance of recovery where the intestine was so much discoloured. The abstinence from food and medicine, and the timely depletion, undoubtedly contributed to this. Most fortunately the inflammatory action was bounded by adhesions, which limited the extent of the abscess to the immediate vicinity of the original wound.

*George-street; June 13th, 1827.*

